# Prevalence of clinically validated primary causes of end-stage renal disease (ESRD) in a State Capital in Northeastern Brazil

**DOI:** 10.1590/2175-8239-JBN-3781

**Published:** 2018-05-17

**Authors:** Luana Rodrigues Sarmento, Paula Frassinetti Castelo Branco Camurça Fernandes, Marcelo Ximenes Pontes, Daniel Barros Santos Correia, Victhor Castelo Branco Chaves, Cecília Ferreira de Araújo Carvalho, Tiago Lima Arnaud, Matheus Henrique Seixas dos Santos, Livia Cristina Barros Barreto, Larissa Alves Alexandre Moliterno

**Affiliations:** 1 Universidade Estadual do Ceará, Fortaleza, CE, Brasil.

**Keywords:** renal insufficiency, chronic, kidney failure, chronic, epidemiology, validation studies, insuficiência renal crônica, falência renal crônica, epidemiologia, estudos de validação

## Abstract

**Introduction:**

Knowledge of validated primary causes of end-stage renal disease (ESRD) is extremely relevant in the realm of public health. The literature lacks validated studies on the primary causes of ESRD.

**Objective:**

The aim of this study was to estimate the prevalence of the causes of ESRD in a State Capital in Northeastern Brazil.

**Methods:**

This cross-sectional study was based on the analysis of medical records of patients on hemodialysis at five specialized centers in Fortaleza, CE, Brazil. Deaths and patients referred to other centers outside Fortaleza were excluded from the study. The data of 830 patients were initially collected, but 818 remained enrolled after the exclusion criteria were applied, the equivalent to 48% of the patents on dialysis in the city.

**Results:**

61.1% of the patients were males. Twenty-two percent of all enrolled individuals were aged 60-69 years. Patient mean age was 55.7 ± 16 years. The most common validated cause of ESRD was unknown (35.3%), followed by diabetes mellitus (26.4%), adult polycystic kidney disease (6.2%), graft failure (6.2%), obstructive uropathy (5.7%), and primary glomerulonephritis (5.3%). Before validation, primary hypertension was the most frequent cause of chronic kidney disease (22.9%), decreased to 3.8% after validation.

**Conclusion:**

The data contradicted national studies reporting primary hypertension as the main cause of chronic kidney disease (CKD). A high rate of unknown causes and categorization bias were observed mainly in relation to primary hypertension as a cause of CKD, which affects the overall prevalence of causes of ESRD in patients on dialysis.

## RESUMO

**Introdução:**

O conhecimento das causas primárias, validadas, de doença renal crônica terminal (DRCT) é primordial no contexto epidemiológico da doença. Existe uma lacuna na literatura em termos de estudos validados sobre tais causas.

**Objetivo:**

Estimar a prevalência das causas de DRCT em uma capital do Nordeste brasileiro.

**Métodos:**

Estudo transversal baseado na análise dos prontuários de pacientes em hemodiálise de cinco centros especializados em Fortaleza, CE. Foram excluídos casos de óbito no período da coleta e de transferências para outras unidades fora do município em questão. Coletou-se dados de 830 pacientes, restando 818 após aplicação dos critérios de exclusão, o correspondente a 48% dos pacientes que dialisam na cidade.

**Resultados:**

Observou-se que 61,1% dos pacientes eram do sexo masculino. A faixa etária mais prevalente foi 60 a 69 anos, 22%. A idade média foi 55,7 ± 16 anos. A causa mais comum de DRCT pós-validação foi indeterminada, 35,3%; seguida por diabetes mellitus, 26,4%; doença renal policística do adulto, 6,2%; falência do enxerto, 6,2%; uropatia obstrutiva, 5,7%; e glomerulonefrite primária, 5,3%. Antes da validação, a hipertensão primária foi a causa mais frequente de DRCT, com 22,9%, e, após validação, caiu para 3,8%.

**Conclusão:**

Os dados contrariam estudos nacionais que afirmam que a primeira causa de DRCT seria hipertensão primária. Evidenciou-se alta taxa de causas desconhecidas e viés de classificação, principalmente com relação à HAS primária como causa de DRCT, o que afeta a prevalência geral das causas de DRCT dos pacientes em diálise.

## INTRODUCTION

The impact of chronic kidney disease (CKD) in mortality, quality of life, and cost of care has increased significantly. Today, 8-16% of the global population is affected by CKD.^1^

CKD is a highly prevalent condition in Brazil. An estimated 11-22 million adult Brazilians - in a country where 70% of the 200 million inhabitants are adults - have some degree of renal impairment. Specialists alone cannot treat this many patients. This is why specific epidemiology programs must be developed and information provided to general practitioners on preventive measures to combat the progression of CKD.^2^^,^ 3

In the Brazilian Northeast, 134 dialysis units - or 18% of the total number of centers in the nation - are registered with the program for individuals with CKD. However, only 55 (41%) joined the Brazilian dialysis census organized by the Brazilian Society of Nephrology (SBN). An estimated 11,308 patients (56.91 pmp) are on hemodialysis in the Brazilian Northeast. Most of the active dialysis centers in Brazil are located in the Southeast; 42% (157) of the 350 units in the region participated in the dialysis survey. The mean national response rate was 38%, and the Northeast was slightly above the Brazilian average.^4^

Data from the Municipal Secretary of Health of Fortaleza from 2015 showed that primary diagnosis was not reported in almost all (97.07%) municipal records of nephrology procedures, since sharing this information is not mandatory.^5^

The prevalence of patients with end-stage renal disease (ESRD) on dialysis increased by 46.8% between 2000 and 2012 - an annual increase of 3.6% - and incidence grew by 20% - or 1.8% a year. There still is considerable uncertainty around the incidence and prevalence of patients with CKD on dialysis in Brazil.^6^

CKD has been underdiagnosed and treated inadequately. Consequently, opportunities to implement primary, secondary, and tertiary preventive care have been missed, partly on account of lack of knowledge on the definitions and categorization of the stages of the disease, and partly because of the limited use of simple tests to diagnose and assess individuals with the disease.^7^

Accurately determining the etiology of ESRD is hindered by the fact that patients often present with atrophic or reduced-size kidneys at the time of diagnosis. The development of preventive strategies and definition of prognostic factors rely on knowledge of the causes of CKD. Despite numerous efforts to collect data on ESRD in Brazil, the country still lacks a national registry with reliable epidemiological data.

This study aimed to estimate the prevalence of causes of ESRD based on careful analysis of the findings by a specialist using strict validation criteria. There is no report in the literature of a similar study carried out in our State.

Studies examining the epidemiology of ESRD may be used to inform the distribution of resources in the area of healthcare and support decisions to further improve patient care and preventive strategies to tackle the disease.^5^

## METHODS

This cross-sectional study was carried out in five of the ten dialysis clinics of Fortaleza, CE, Brazil, from January to June f 2016.

According to the National Registry of Healthcare Units (Cadastro Nacional de Estabelecimentos de Saúde - CNES), the ten hemodialysis clinics in Fortaleza serve about 1,700 individuals with CKD.^8^

The clinics were selected so that at least one unit belonging to each Regional Executive Secretariat (SER) was included in the study. The SER functions as a sort of “sub-prefecture,” whose role includes managing infrastructure, basic sanitation, and healthcare services, to name a few. The selected units were chosen from a list containing all dialysis units, to include the clinics with greater number of patients assigned to each SER, so that all districts of the municipality were included and the sample was not biased for socioeconomic status. Two of the six SERs did not have clinics. Therefore, four units were included in the study.

The units included in the study, as almost all clinics in the municipality, were privately held and managed by groups of nephrologists with the exception of one, a non-profit teaching clinic belonging to the hospital of a federal university. All included units were registered with the Brazilian Public Healthcare System (SUS).

The studied population comprised patients with CKD on dialysis at the time of the study. Included individuals had to meet the following enrollment criteria: patients on dialysis (for more than three months) for ESRD at the time of the study with care funded by the SUS. The medical records of the patients who died during the study and of patients referred to other dialysis units outside Fortaleza were excluded. The patients were not interviewed. The authors went to the dialysis units to collect information from the charts (paper and electronic records) of the patients included in the study.

Data from 830 patient charts - approximately 48.82% of the patients with CKD on dialysis in the municipality - were collected and analyzed. The application of standardized criteria^9^ decreased the sample size to 818 patient charts - 48.18% of the patients with CKD on dialysis in the municipality.

### DATA COLLECTION PROCEDURES

The data were collected from a secondary source (patient charts). In the first stage of the study, a pilot-test was run with 20 patient charts to test the data collection form, train the students helping with data collection, and standardize the information collected. The clinics sent an updated list with active patients registered with their dialysis programs, from which the authors drew a preliminary list after applying the enrollment criteria. The exclusion criteria were then applied to the preliminary list before the final list was produced.

In the second stage of the study, one specialist - nephrologist PFCBCF - validated the diagnoses. Having one specialist analyzing the records decreased bias and intra-observer variability.

### DATA COLLECTION FORM

The form used in this study for purposes of data collection was designed and validated, and the guiding diagnostic criteria were defined. Three nephrologists, an epidemiologist, and a nurse designed the data collection form and defined the diagnostic criteria.^9^

The data collection form was divided into the following sections: patient history and clinical findings; workup and imaging; and histopathology. The purpose was to determine the primary cause of renal disease (see attached document). The diagnosis of primary kidney disease cited in the patient charts with the corresponding codes from the 10^th^ revision of the International Statistical Classification of Diseases and Related Health Problems (ICD-10) were recorded in the form.

### ETHICAL ASPECTS

The study was approved by the Ethics Committee (Plataforma Brasil) and given permit no. 19989414.3.0000.5534. Each clinic signed a custody term.

## RESULTS

The causes of ESRD present in 818 patient charts were analyzed and validated. Male patients accounted for 61.1% of the studied sample. The age ranges were divided into percentiles. The most prevalent range was from 60 to 69 years of age, with 22% (180) of the patients. The mean age was 55.7 ± 16 years, with values ranging from 18 to 94 years. Mean time on dialysis was 7 ± 6.1 years. The standard deviation was quite broad, since patients had been on dialysis for one to 33 years. The calculation of the median time on dialysis revealed that 50% of the patients had been treated for less than five years; percentile analysis showed that 75% of the patients had been treated for fewer than ten years (Table 1).

**Table 1:** Prevalence of causes of ESRD in Fortaleza, CE, Brazil, before and after validation (2014-2016)

Cause	Before		After	
	n	%	n	%
Undetermined cause	287	35.1	316	38.6
Diabetes mellitus	148	18.1	219	26.7
Adult polycystic kidney disease	46	5.6	53	6.4
Obstructive uropathy	25	3.1	48	5.8
Primary glomerulonephritis	45	5.5	48	5.8
Secondary glomerulonephritis	19	2.3	32	3.9
Primary hypertension	187	22.9	40	4.8
Chronic pyelonephritis	16	2.0	22	2.7
Inherited kidney disease	6	0.7	9	1.1
Chronic interstitial nephritis	10	1.2	9	1.1
Gestational hypertension	-	-	4	0.5
Secondary hypertension	15	1.8	4	0.5
Renovascular hypertension	-	-	2	0.2
Congenital nephropathy	1	0.1	-	-
Other	5	0.6	7	0.9

The most prevalent primary causes of ESRD were unknown (38.6%); diabetes mellitus (26.7%); glomerulonephritis (9.7%); adult polycystic kidney disease (PKD) (6.4%), obstructive uropathy (5,8%); and primary hypertension (5%).

Primary hypertension was ranked second among the more common causes of ESRD with 22.9% (n=187) of the patients, but after validation it moved to the eighth spot with 3.8% (n=31). According to the SBN census (2015), primary hypertension was listed as the main cause of ESRD from 2011 to 2015.

Graft failure was included as a secondary cause of ESRD to reflect the rate of return to dialysis after graft loss, given that this information is scarce in Brazil (Table 2). Graft failure was seen in 6.2% (n=51) of the cases after validation.

**Table 2:** Primary causes of ESRD after validation in patients with failed grafts, Fortaleza, CE, Brazil (2016)

Cause	Post-validation
Undetermined cause	27
Diabetes mellitus	3
Primary hypertension	9
Secondary hypertension	1
Primary glomerulonephritis	5
Inherited kidney disease	2
Obstructive uropathy	1
Adult polycystic kidney disease	2

The disagreement between the diagnoses listed in the patient charts versus the validated diagnoses was 39.6%. Disagreement between diagnoses was observed when the diagnostic ICD codes (as listed in the charts) were compared to the outcome of the analysis of the forms performed by the authors, revealing a significant level of categorization bias, i.e., discordant records of causes of CKD after validation.

Emergency hemodialysis (HD) was performed in 69.1% (279/404) of the patients. The proportion becomes even more significant when it is interpreted vis-à-vis the primary diagnosis: 76.6% (105/137) of the patients with disease caused by unknown factors underwent emergency HD.

## DISCUSSION

The study revealed a higher prevalence of CKD among male individuals, as reported by Sesso et al. (2014) and Banaga et al. (2015). The predominance seen in the group aged 60-69 years is in agreement with the mean age of the population with CKD in Brazil.^10^

A study on the clinical/epidemiological profile of individuals on chronic HD in João Pessoa, PB, Brazil, reported that systemic hypertension was the most prevalent etiology of CKD with 94 cases (38%), and diabetes mellitus (DM) the second with 32 cases (13%). Twenty-four patients (10%) were assumed to have the two conditions as causes of disease, without proof or validation. Sixty-eight patients (28%) had unknown CKD etiology.^11^

Another Brazilian study listed systemic hypertension as the first cause of CKD (41.2%) followed by DM (32.4%), uropathy (11.2%), chronic glomerulonephritis (5.6%), and graft loss (0.7%). Unknown primary cause of disease was seen in 7.7% of the cases.^12^ The studies mentioned above did not describe the diagnostic criteria for baseline disease and did not state whether any clinical validation was performed. This means that the cause of disease was assumed, and not proven with the support of diagnostic criteria; for example, the authors did not state whether hypertension was a cause or consequence of CKD.

According to the United States Renal Data System (USRDS) (2015), the North-American reference in dialysis registries, before 1997 the more common primary diagnosis for incident patients with ESRD in the USA had been glomerulonephritis. However, since 1997 the number of patients starting dialysis having diabetes as the causing condition exceeded the number of individuals with glomerulonephritis (incident patients). The prevalence of diabetes and hypertension as primary diagnoses of ESRD has increased considerably. Diabetes overtook glomerulonephritis in 2011 and became the most common primary cause when prevalence data is considered.^13^

The change was confirmed in Europe in the 2015 census survey based on data from 2013. The institution organizing the survey, the *European Renal Association - European Dialysis and Transplant Association* (ERA-EDTA) (2015), informed that diabetes mellitus was the cause of kidney failure with the highest incidence in Europe (22.4%) in 2013, followed by unknown cause (17.1%), other causes (17.1%), and hypertension (15.2%). In the same year, diabetes mellitus (22.2%), other causes (17.1%), glomerulonephritis/glomerulosclerosis (16.6%), and undetermined causes (14.3%) were the etiologies with higher incidence among individuals aged 65 years or younger. Hemodialysis (79.2%) was the mode of treatment with the highest incidence, followed by peritoneal dialysis (15.0%), and kidney transplant (5.7%).^14^

There is abundant evidence suggesting that most screening programs tend to further evince inequalities, instead of correcting them. This is particularly true when they are carried out in countries where the public healthcare system is insufficiently organized and/or when screening programs are not organized by the government, thus producing the so-called “opportunistic screening programs.” This is almost always the case in places where the public healthcare system is disorganized and private healthcare is stronger.^15^

### STUDY LIMITATIONS

The study pointed out weaknesses in the quality of the patient records when it came to determining the baseline disease. A significant amount of relevant information was not in the records, and some were found in separate lists or folders. The great proportion of undetermined causes of disease may affect the order of importance of causes of ESRD.

A strength of the study was the fact that it was a pioneering initiative in the State of Ceará and in Brazil, as it collected data from 830 patients with ESRD - 48% of the patients in the municipality - to identify the causes of chronic kidney disease in patients on dialysis based on the best available evidence.

Establishing the diagnosis of primary kidney disease based on clinical criteria is no simple task, even when the existing evidence is systematically and thoroughly analyzed, particularly in regards to essential hypertension as a cause of ESRD. Clinical syndrome “hypertensive kidney disease” is still a poorly defined condition. The relationship between moderate hypertension, nephrosclerosis, and ESRD is still unclear, despite the ongoing clinical and experimental trials. The answers might involve categorization bias, environmental factors, and genetic predispositions.

## CONCLUSION

The most frequent cause of ESRD found in this study was diabetes. Primary hypertension as a cause of ESRD was overestimated. The diagnosis of the primary causes of ESRD should be based on standardized criteria periodically revised and updated at dialysis units and reported to the State Secretaries of Health.

Emergency dialysis was offered to 69.1% of the patients, a reflection of the troubles inherent to identifying the baseline disease - given that the patients had advanced disease - and of the difficulties patients experience with access to healthcare services.

Participation in the editions of the Brazilian dialysis census is of the utmost importance and should be mandatory, given the wealth of information and contributions it offers to the development of national registries and the comparisons it allows between Brazilian States and against other regional registries, in addition to facilitating the analysis of trends in CKD and renal replacement therapy in the nation.

In a context of increasing prevalence and incidence of CKD, epidemiology studies examining aspects connected to prevention and treatment of CKD are exceedingly relevant. Knowledge of the baseline diseases causing CKD is very important in the development of public policies for populations at risk devised to diagnose patients and establish strategies to prevent and delay the progression of CKD.

## SUPPLEMENTARY MATERIAL

The following document is available online:

Annex
