# Supplementary material for: Prevalence of clinically validated primary causes of end-stage renal disease (ESRD) in a State Capital in Northeastern Brazil
Source: J Bras Nefrol. 2018 May 17;40(2):130–5. doi: 10.1590/2175-8239-JBN-3781 (PMC6533992; doi:10.1590/2175-8239-JBN-3781)
Supplement: Supplementary file 1 [file 2175-8239-jbn-3781-suppl.pdf]

Supplementary Material from “Prevalence of clinically validated primary causes of end-stage renal disease (ESRD) in a State Capital in Northeastern Brazil”

ANNEX

FORM TO EXAMINE THE CAUSES OF ESRD

I IDENTIFICATION

1 Record number (patient chart) _____________________

Name ____

1.1 Date of birth ____/____/____

1.2 Sex:

1. Male ( )   2. Female ( )

1.3 Dialysis start date at the unit: ____/____/____

II DIAGNOSIS

2 Primary disease listed on the patient chart: ________________________

2.1 ICD-10: ________________

III BASIS FOR DIAGNOSIS:

<u>Clinical factors</u>:

3 Nephrotic syndrome: 0 No ( ) 1 Yes ( ) 2 Unknown ( )

4 Urolithiasis: 0 No ( ) 1 Yes ( ) 2 Unknown ( )

5 Prolonged use of nephrotoxic drugs:

0 No ( )   1 Yes ( )   2 Unknown ( )   3 Not applicable ( )

5.1 Drug name(s) ____

6 Prolonged use of illegal drugs:

0 No ( )   1 Yes ( )   2 Unknown ( )   3 Not applicable ( )

6.1 Specify: ____

7 Obstructive urologic symptoms:

0 No ( )   1 Yes ( )   2 Unknown ( )   3 Not applicable ( )

7.1 Other factors: ____

8. Is there a family history of kidney disease?

0 No ( )   1 Yes ( )   2 Unknown ( )   3 Not applicable ( )

8.1 Specify: ____

A. Imaging:

9. Ultrasound:

0 No ( )   1 Yes ( )   2 Unknown ( )

9.1 If yes, kidney size:

0 normal ( )   1 decreased ( )   2 unknown ( )   4 normal ( )

9.2 1 L kidney ______ 2 R kidney ______ 3 Not applicable ( )

9.3 OTHER (MRI, CT) ________________________

10 Kidney biopsy:

0 No ( ) 1 Yes ( ) 2 Unknown ( )

If yes:

10.1 Date of biopsy: ______________

10.2 Specify: ____

2 Unknown ( ) 3 Not applicable ( )

11 Antibodies

11.1 ANCA 0 No ( ) 1 Yes ( ) 2 Unknown ( )

11.2 ANA 0 No ( ) 1 Yes ( ) 2 Unknown ( )

11.3 DNA No ( ) 1 Yes ( ) 2 Unknown ( )

11.4 Outros No ( ) 1 Yes ( ) 2 Unknown ( )

11.4.1 Specify: ____

2 Unknown ( ) 3 Not applicable ( )

11.5 Protein electrophoresis: No ( ) 1 Yes ( ) 2 Unknown ( )

11.6 Angiogram No ( ) 1 Yes ( ) 2 Unknown ( )

11.6.1 Result (arteriogram/MRA): ____________________________

2 Unknown ( ) 3 Not applicable ( )

B. Hypertension/Diabetes:

12 Treatment for hypertension 0 No ( ) 1 Yes ( ) 2 Unknown ( )

12.1 Highest systolic BP on record ____________ 2 Unknown ( )

12.2 Highest diastolic BP on record ____________ 2 Unknown ( )

12.3 Is there evidence of accelerated hypertension (grade III/IV retinopathy)?

0 No ( ) 1 Yes ( ) 2 Unknown ( )

13 Is/was the patient diabetic? 0 No ( ) 1 Yes ( ) 2 Unknown ( )

IF YES, if NO, score as: 3 Not applicable ( )

13.1 1 DM type I ( ) 2 DM type II ( ) Other ________________

13.2 Age at diagnosis (years): _______________

13.3 Has the patient been treated with insulin? 0 No ( ) 1 Yes ( ) 2 Unknown ( )

13.4 Time diagnosed with DM at the start of insulin therapy (years)

____________ 2 Unknown ( )

14 Was there proteinuria? 0 No ( ) 1 Yes ( ) 2 Unknown ( )

14.1 Maximum level: _________ 2 Unknown ( ) 3 Not applicable ( )

14.2 Was there macroscopic hematuria? 0 No ( ) 1 Yes ( ) 2 Unknown ( )

14.3 Was there microscopic hematuria? 0 No ( ) 1 Yes ( ) 2 Unknown ( )

15 Was there any evidence of target-organ injury?

0 No ( ) 1 Yes ( ) 2 Unknown ( ) 3 Not applicable ( )

Microvascular	Retinopathy ( )	Hypertension ( )		Diabetes ( )	
		GI-II ( )	GIII-IV ( )	N-prolif ( )	Prolif. ( )
	Neuropathy ( )			Not specified ( )	
Macrovascular	Cerebrovascular	Stroke ( ) Hemorrhagic stroke ( )		TIA ( ) Stroke ( )	
	Coronary	AMI ( ) Angina ( )		Revascularization ( )	
	Peripheral vascular	Grafts ( )		Amputations ( )<br>RLL ( ) LLL ( ) Toe ( ) Other ( )	

C. Supplementary information

16 Day when the patient first presented with kidney disease ___/___/___

2 Unknown ( )

17 Day when creatinine > 1.0 (fem)/1.3 (male) for the first time ___/___/___

2 Unknown ( )

18 Day when the patient was first referred to a nephrologist ___/___/___

2 Unknown ( )

19 Day of first renal replacement therapy session ___/___/___

2 Unknown ( )

20 Has the patient ever undergone emergency hemodialysis?

0 No ( )   1 Yes ( )   2 Unknown ( )

21 Creatinine level at first renal replacement therapy session: ___________

0 No ( )   2 Unknown ( )

D. Summarized baseline disease diagnosis:

____

____

22. Diagnosis**:** 0 No ( ) 2 Unknown ( ) 3 Not applicable ( )

22.1 Primary: _________________________

22.2 ICD-10: __________

23 Comorbidities: ( )

23.1.1 ICD-10: __________

23.2.1 ICD-10: __________

24. Did the diagnosis in the patient chart agree with the validated diagnosis?

0 No ( ) 1 Yes ( )

25. Validated diagnosis 0 No ( ) 1 Yes ( ) 3 Not applicable ( )

If not:

25.1 Primary: _________________________

25.2 Comorbidities: ____________________

25.2.1 ICD-10 ___________
